# Troponin T-release associates with cardiac radiation doses during adjuvant left-sided breast cancer radiotherapy

**DOI:** 10.1186/s13014-015-0436-2

**Published:** 2015-07-10

**Authors:** Tanja Skyttä, Suvi Tuohinen, Eeva Boman, Vesa Virtanen, Pekka Raatikainen, Pirkko-Liisa Kellokumpu-Lehtinen

**Affiliations:** Department of Oncology and School of Medicine, Tampere University Hospital, University of Tampere, 33521 Tampere, Finland; Heart Center Co and School of Medicine, University of Tampere, Tampere University Hospital, Tampere, Finland; Department of Oncology, Tampere University Hospital and Department of Medical Physics, Medical Imaging Center and Hospital Pharmacy, Tampere, Finland; Department of Medicine, Central Finland Health Care District and University of Eastern Finland, Jyväskylä, Finland

**Keywords:** Radiotherapy, Breast cancer, Cardiotoxicity, High sensitivity cardiac troponin T

## Abstract

**Background:**

Adjuvant radiotherapy (RT) for left-sided breast cancer increases cardiac morbidity and mortality. For the heart, no safe radiation threshold has been established. Troponin T is a sensitive marker of myocardial damage. Our aim was to evaluate the effect of left-sided breast cancer RT on serum high sensitivity troponin T (hscTnT) levels and its association with cardiac radiation doses and echocardiographic parameters.

**Methods:**

A total of 58 patients with an early stage, left-sided breast cancer or ductal carcinoma in situ (DCIS) who received adjuvant breast RT without prior chemotherapy were included in this prospective, non-randomized study. Serum samples were taken before, during and after RT. An increase of hscTnT >30 % was predefined as significant. A comprehensive 2D echocardiograph and electrocardiogram (ECG) were performed before and after RT. Dose-volume histograms (DVHs) were generated for different cardiac structures.

**Results:**

The hscTnT increased during RT from baseline in 12/58 patients (21 %). Patients with increased hscTnT values (group A, *N* = 12) had significantly higher radiation doses for the whole heart (*p* = 0.02) and left ventricle (*p* = 0.03) than patients without hscTnT increase (group B, *N* = 46). For the left anterior descending artery (LAD), differences between groups A and B were found in volumes receiving 15 Gy (*p* = 0.03) and 20 Gy (*p* = 0.03) Furthermore, after RT, the interventricular septum thickened (*p* = 0.01), and the deceleration time was prolonged (*p* = 0.008) more in group A than in group B.

**Conclusions:**

The increase in hscTnT level during adjuvant RT was positively associated with the cardiac radiation doses for the whole heart and LV in chemotherapy-naive breast cancer patients. Whether these acute subclinical changes increase the risk of excessive long-term cardiovascular morbidity or mortality, will be addressed in the follow-up of our patients.

## Background

Postoperative radiotherapy (RT) for breast cancer is an essential part of adjuvant cancer treatment. RT reduces the risk of local recurrence by 50 % and the risk of breast cancer mortality by 16 % [1]. However, left-sided RT, especially, has been shown to induce excess cardiovascular mortality and morbidity [1–4]. In a retrospective study, Darby et al. showed that the risk of major cardiac events in patients with left-sided breast cancer increased by 7.4 % for each increase of 1 Gy of radiation to the heart. Furthermore, prior cardiovascular diseases, such as hypertension, may further increase the risk of radiation induced cardiac damage [4].

Radiation induced cardiac changes have been demonstrated with echocardiographic strain rate imaging [5], myocardial perfusion scintigraphy [6, 7] and single photon emission tomography [8]. In contrast, no changes in the left ventricle (LV) systolic function were detected in basic echocardiographic measurements [5, 7]. Majority of patients in these studies also received prior or concurrent chemotherapy [5–8], which is known to be associated with cardiotoxicity [9, 10]. High sensitivity cardiac troponin T (hscTnT) is able to detect minor myocardial damage during RT [5, 11]. The mechanism of this acute damage is thought to evolve from negative changes in the microvasculature during RT [12].

In this study, we included only chemotherapy-naive breast cancer patients to exclude chemotherapy-induced prior cardiotoxicity. The patients were studied prospectively using a cardiac biomarkers, comprehensive 2D echocardiography and 12-lead electrocardiogram (ECG) before and after adjuvant RT to detect subclinical cardiac changes. Furthermore, dose volume histograms (DVHs) for different cardiac structures were generated and correlated with serum markers and echocardiographic measurements.

## Methods

### Patient population

This single centre, prospective, observational clinical study included 60 eligible female patients with early stage left-sided breast cancer or DCIS. All patients were treated with adjuvant conformal 3D RT after breast conserving surgery (*n* = 59) or mastectomy (*n* = 1) without axillary or supraclavicular lymph node RT. Two patients were excluded from final analysis due to missing serum samples. Patients with prior adjuvant chemotherapy were excluded. Other exclusion criteria were age over 80 years, dialysis, recent acute myocardial infarction, symptomatic heart failure, chronic atrial fibrillation, pacemaker therapy and severe lung disease. The local ethical committee approved the protocol and all participants signed informed consent before study enrollment. The study was conducted from June 2011 to May 2013.

### Radiation therapy

All patients had 3D computer tomography (CT) treatment planning with Philips Big Bore CT (Phillips Medical Systems, Madison, WI, USA) or Toshiba Aquilion LB (Toshiba Medical System, Tokyo, Japan) on a breast board in the supine position with their arms above their heads. CT slices (3 mm thickness) were used without intravenous contrast. Scanning was performed during free breathing in 56 patients, whereas 2 patients were scanned and treated using the voluntary deep inspiration breath hold (vDIBH) technique, which was implemented in our unit in April 2013 as part of clinical practice. In vDIBH, the breathing cycle was monitored with Varian real time position management (RPM) system (Varian Medical Systems, Palo Alto, CA, USA). Treatment contouring and planning were done with Eclipse v.10 system (Varian Medical Systems, Palo Alto, CA, USA). Planning target volume (PTV) covered the remaining breast tissue in 57 patients and the chest wall in one patient (mastectomy) with sufficient margins to account for the inter- and intrafraction movements (5–8 mm in our unit).

Treatment doses were either 50 Gray (Gy) in 2 Gy fractions with or without additional boost (10–16 Gy, 5–8 fractions) to tumor bed or 42.56 Gy in 2.66 Gy fractions (hypofractionation) over 3.5 weeks. Tangential photon fields were used in 57 patients and chest wall in one patient with mastectomy was treated with electron beams. Doses were calculated with anisotropic analytical algorithm (AAA v.10, Varian Medical Systems, Palo Alto, CA, USA) for photons and Electron Monte Carlo algorithm (eMC v.11, Varian Medical Systems, Palo Alto, CA, USA) for electrons. Dose volume histograms (DVH) of different structures were generated for each patient. To account for the different dosing schedules, an α/β-ratio of 3 was used for heart and lung to calculate 2 Gy equivalent doses.

The heart, LV and LAD were contoured from the treatment planning CT scans as suggested by Feng et al. [13]. All cardiac structures were contoured by the same radiation oncologist (TS).

### Serum biomarker analysis

High sensitivity cardiac troponin T (hscTnT, ng/l) and B-type natriuretic peptide (BNP, ng/l) were analyzed from serum samples taken before, after two (hypofractionated RT) or three (conventional RT) weeks of treatment and at the last day of RT. As the lowest detection limit of hscTnT was 5 ng/l, the values below this (<5 ng/l) were estimated to be 4 (lowest detection limit (LOD)/√2) when calculating the percentage increase from the baseline value [14]. A predefined increase of >30 % from the baseline was considered to be clinically important according to the study protocol. The patients were divided into two groups based on their hscTnT change: group A with a hscTnT increase more than 30 % from baseline during RT and group B without a hscTnT increase.

Total cholesterol levels (mmol/l) were measured at baseline under fasting conditions.

### Echocardiographic examinations

A comprehensive echocardiography study and 12-lead ECG were performed at the baseline and at the completion of RT. All echocardiography examinations were performed using the same ultrasound machine (Philips iE33 ultrasound system, Bothell, WA, USA) and a 1–5 MHz matrix-array X5-1 transducer by the same cardiologist certified by European Association of Echocardiography for adult transthoracic echocardiography. All images were acquired at rest. Subcostal imaging was performed in the supine position, while other imaging was performed with the patient in the lateral decubitus position with simultaneous superimposed ECG. Doppler recordings were acquired at the end of expiration during shallow breathing. Images were stored digitally for use with offline analysis software (Excelera, Philips, Koninklijke, Netherlands; Qlab, Philips, Bothell, USA). Echocardiographic measurements were performed in a standardized manner according to the European guidelines [15].

### Statistical analysis

Data are expressed as means ± standard deviation (SD) for normally distributed continuous variables and as medians with inter quartile range (IQR) for variables with non-normal distributions. The study groups were compared using the *t*-test for independent samples or the Mann–Whitney *U* test. Friedman’s analysis of variance was used for repeated measures of non-normal variables and the Wilcoxon signed ranks test was used to compare the hscTnT before RT vs. hscTnT after RT. Categorical data are expressed as numbers (%) of subjects. The Chi-squared test was used for categorical variables and the Fisher’s exact test was used when appropriate. The related samples Cochran’s Q test was used to study the change in dichotomous variables measures more than twice. Area under curve (AUC) was calculated using the trapezium rule in order to summarize the information from DVH. All the tests were two-sided and a p value <0.05 was considered statistically significant. Analysis was performed using IBM SPSS Statistics for Windows (version 21.0, Armonk, NY, USA, IBM Corp.).

## Results

Baseline characteristics of the patients are presented in Table 1. The baseline variables (e.g. age, body mass index, cholesterol and hormonal use) were similar between the two groups A and B based on the troponin increase. However, in the group A patients tended to have more hypertension (*p* = 0.31) and less thyroid hormone supplementation (*p* = 0.19).

**Table 1:** Patient baseline characteristics. Results are presented as mean ± SD for normally distributed continuous variables, median (inter-quartile range) for non-normal variables and n (%) for categorical variables

	All (*N* = 58)	Group A (*N* = 12)	Group B (*N* = 46)	*p*-value*
Age	63 ± 6	65 ± 6	63 ± 7	0.37
BMI, kg/m^2^	27 ± 4	27 ± 5	27 ± 4	0.76
Hypertension	20 (34 %)	6 (50 %)	14 (30 %)	0.31
ACE or ARB	14 (24 %)	4 (33 %)	10 (22 %)	0.46
Beta-blocker	7 (17 %)	3 (25 %)	4 (9 %)	0.15
Calcium channel blocker	4 (7 %)	2 (17 %)	2 (4 %)	0.19
CAD	1 (2 %)	0 (0 %)	1 (2 %)	1.00
Statin use	13 (22 %)	3 (25 %)	10 (22 %)	1.00
Diabetes	4 (7 %)	1 (8 %)	3 (7 %)	1.00
Hypothyreosis	8 (14 %)	0 (0 %)	8 (17 %)	0.19
Current smoking	8 (14 %)	2 (17 %)	6 (13 %)	0.66
Hormonal therapy	23 (40 %)	4 (33 %)	19 (41 %)	1.00
AI	22 (38 %)	5 (42 %)	17(37 %)	0.75
Tamoxifen	2 (3 %)	0 (0 %)	2 (4 %)	1.00
Troponin T, ng/l	4 (4–6)	4(4–4.5)	4 (4–7)	
Cholesterol, mmol/l	5.6 ± 1.1	5.5 ± 0.9	5.6 ± 1.1	0.90
BNP, ng/l	58 (37–124)	78 (33–123)	58 (41–125)	0.72

*BMI* body mass index, *ACE* angiotensin converting enzyme, *ARB* angiotensin II receptor blockers, *CAD* coronary artery disease, *Statin use* hypercholesterolemia with statin use, *Diabetes* blood glucose lowering diabetic medication, *ASA* low dose daily acetylsalicylic acid, *Hypothyreosis* continuous thyroid hormone supplementation, *AI* aromatase inhibitor, *BNP* b-type natriuretic peptid

* Independent samples *t*-test was used for normally distributed variables and Mann–Whitney *U* test for non-normal variables; Fisher’s exact test was used for categorical variables

### High sensitivity cardiac troponin T

In the whole study population, hscTnT was detectable (≥5 ng/l) in 25 (43 %) patients before RT, in 28 (48 %) at 3 weeks and in 30 (52 %) patients after RT. The serum samples after RT were taken at the last day of RT (median 0, range −1 − +3 day).

**Fig. 1:**
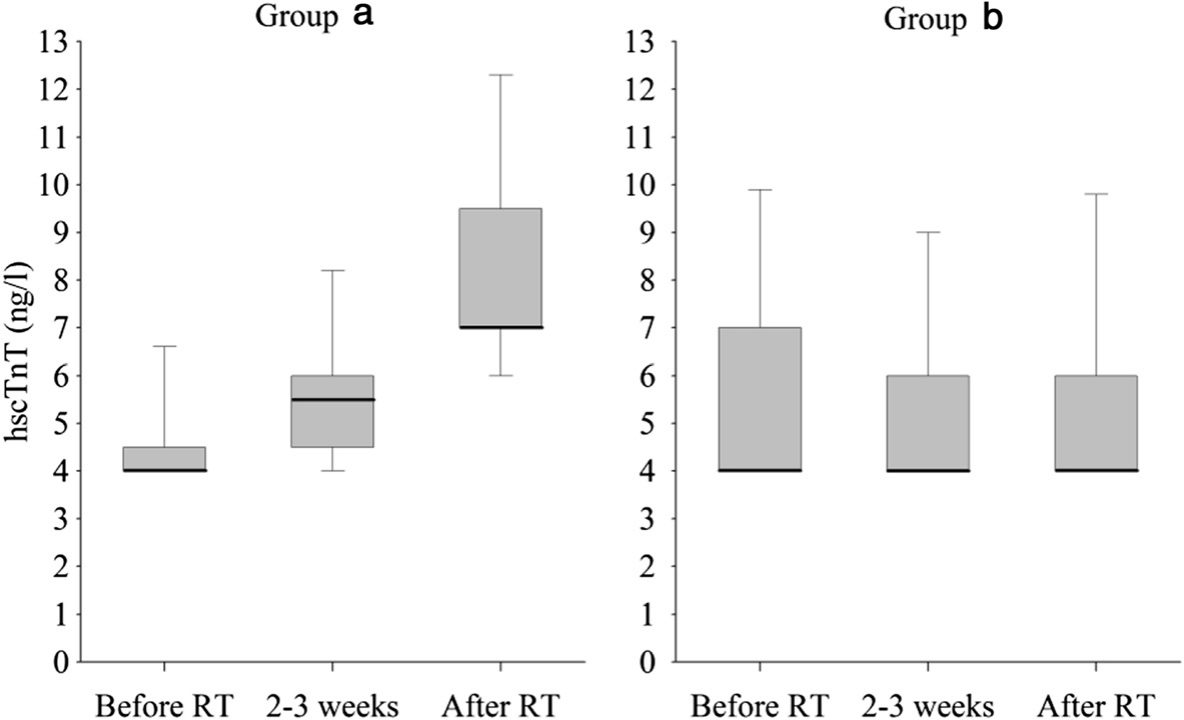
Box plot figures to describe high sensitivity cardiac troponin T (hscTnT) before RT, at weeks 2–3 and after RT in patients with increased hscTnT release (Group **a**, *N* = 12) and in patients without hscTnT release (Group **b**, *N* = 46). Borders of the box represent the upper and lower quartiles, bold lines the medians and error bars above and below the box represent the 90^th^ and 10^th^ percentiles

Median (IQR) hscTnT for the whole study population was 4 (4–6) ng/l before RT, 4 (4–6) ng/l at 3 weeks and 5 (4–7) ng/l after RT. The difference in hscTnT level before RT and after RT was not significant for the whole population (*p* = 0.08). However, hscTnT increased over 30 % from the baseline value in 12/58 (21 %) patients constituting the group A. In these 12 patients, hscTnT was detectable in 3 (25 %) before RT, in 9 (75 %) at 2–3 weeks and in 12 (100 %) patients after RT. The median (IQR) values at these time points were 4 (4–4.5)ng/l, 5.5 (4.5-6)ng/l and 7 (7–9.5)ng/l, respectively (Fig. 1). In the hscTnT stable group B (*N* = 46,) hscTnT was detectable in 22 (48 %) at the beginning, 19 (41 %) at 2–3 weeks and 18 (39 %) after RT with median (IQR) values of 4(4–7), 4(4–6) and 4(4–6) ng/l, respectively.

### B-type natriuretic peptide

In the whole study population BNP levels were 58 (37–124), 69 (42–135) and 74 (34–126) mmol/l before RT, at 2–3 weeks and after RT, respectively (*p* = 0.72). The BNP change between the first and the last time point was −5 (−26 to 5) mmol/l in the group A vs. 5 (−14 to 22) mmol/l in the group B (*p* = 0.33).

### Cardiac doses

**Fig. 2:**
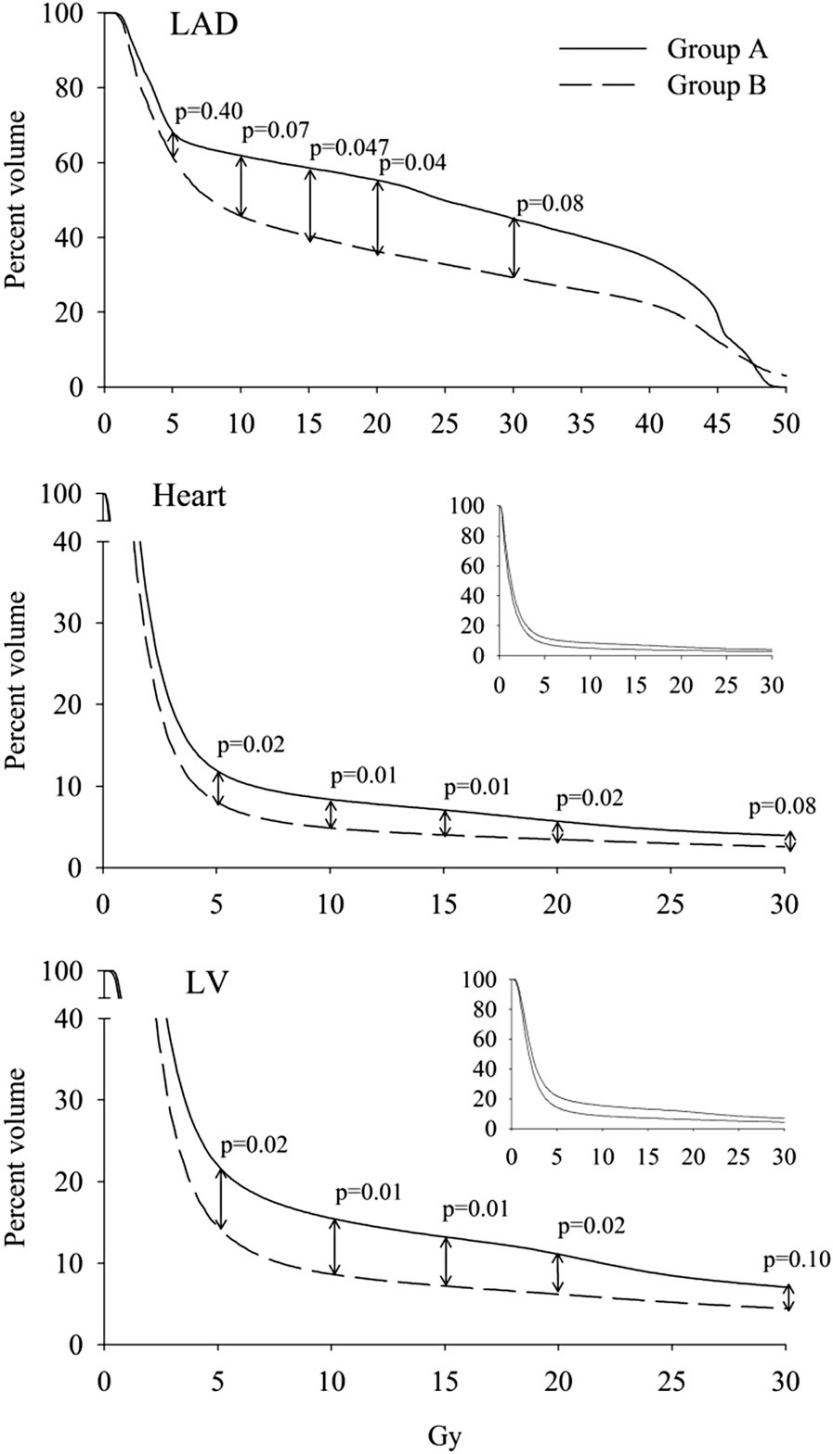
Dose volume histogram (DVH) curves for left anterior descending artery (LAD), heart and left ventricle (LV) in patients with increased high sensitivity cardiac troponin T (hscTnT) release (Group A, *N* = 12) and in patients without hscTnT release (Group B, *N* = 46)

The DVH curves for the both groups are presented in Fig. 2. The AUC for heart and left ventricle was significantly higher in the hscTnT-positive group A compared with group B (*p* < 0.05, Table 2). The same trend was seen for the LAD (*p* = 0.08). In addition to AUC, some relevant dose-volume parameters (volume of structure receiving 5 Gy radiation dose = V5 and similarly V10, V15, V20 and V30) were separately tested (Fig. 2 and Table 2). In the heart and left ventricle, the difference between groups was significant in all those Gy points from 5 to 20 Gy, but in the LAD the difference between groups was significant only at dose levels of 15 and 20 Gy.

**Table 2:** Radiation doses to cardiac structures in patients with hscTnT increase >30 % from baseline during RT (Group A) vs. patients without hscTnT increase (Group B)

Cardiac structure	Group A (N = 12)	Group B (N = 46)	*p* value*
Heart			
Dmean (Gy)	4.0 ± 1.8	2.8 ± 1.3	0.02
Dmax (Gy)	48.9 ± 5.3	43.9 ± 11.0	0.13
V5 (%)	12.0 ± 6.2	8.1 ± 4.6	0.02
V10 (%)	8.4 ± 5.1	4.9 ± 3.4	0.01
V15 (%)	7.1 ± 4.4	4.0 ± 3.1	0.01
V20 (%)	5.7 ± 3.3	3.5 ± 2.8	0.02
V30 (%)	4.0 ± 2.6	2.6 ± 2.3	0.08
AUC (%.Gy)	403 ± 172	291 ± 134	0.02
LV			
Dmean (Gy)	6.7 ± 3.3	4.5 ± 2.6	0.02
Dmax (Gy)	47.8 ± 5.8	41.0 ± 12.2	0.07
V5 (%)	22.0 ± 12.3	14.5 ± 9.2	0.02
V10 (%)	15.5 ± 10.2	8.6 ± 7.0	0.01
V15 (%)	13.2 ± 9.2	7.2 ± 6.4	0.01
V20 (%)	11.1 ± 7.7	6.2 ± 5.9	0.02
V30 (%)	7.1 ± 5.4	4.4 ± 4.7	0.10
AUC (%.Gy)	668 ± 333	468 ± 260	0.03
LAD			
Dmean (Gy)	23.8 ± 10.1	17.5 ± 10.8	0.07
Dmax (Gy)	43.4 ± 11.8	37.9 ± 15.8	0.27
V5 (%)	68.3 ± 24.2	61.7 ± 24.1	0.40
V10 (%)	61.9 ± 26.1	45.7 ± 27.7	0.07
V15 (%)	58.6 ± 26.3	40.0 ± 28.0	0.047
V20 (%)	55.4 ± 26.3	36.2 ± 28.3	0.04
V30 (%)	45.0 ± 25.3	29.3 ± 27.8	0.08
AUC (%.Gy)	2422 ± 1053	1793 ± 1097	0.08

*hscTnT* high sensitivity cardiac troponin T, *RT* radiotherapy, *Dmean* mean radiation dose to the structure, *Dmax* maximal point radiation dose in the structure, *V30/20/10/5* the volume of structure receiving 30Gy/20Gy/10Gy/5Gy dose, *AUC* area under the curve, *LV* left ventricle, *LAD* left anterior descending coronary artery

*Independent samples *t*-test

### Echocardiographic findings

In group A, the increase in hscTnT was accompanied by minor changes in the echocardiographic measurements as presented in Table 3. Measured before and after RT, the interventricular septum thickened (*p* = 0.01) accompanied with a change in LV diastolic function as the deceleration time increased (*p* = 0.008) among group A more than in group B. In other LV diastolic parameters, the mitral E-peak decreased in both groups. The decrease in the LV end diastolic and end systolic diameters in the group A were not statistically significant compared with group B (*p* = 0.06 and *p* = 0.11, respectively).

**Table 3:** Echocardiographic measurements at baseline(before RT) and the change from baseline measured at the end of RT in patients with increased hscTnT release (group A) and in patients without hscTnT release (group B)

	Group A (N = 12)		Group B (N = 46)		
	Before RT	Change	Before RT	Change	P*
LV dimensions					
LVEDD (mm)	44.0 (42.0 - 45.5)	-2.0 (-3.5 - 0.0)	46.0 (44.0 - 47.0)	-0.5 (-2.0 - 2.0)	0.06
LVESD (mm)	30.0 (27.5 - 31.0)	-1.5 (-4.5 - 1.5)	31.0 (29.0 - 33.0)	0.0 (-2.0 - 2.0)	0.11
IVS (mm)	11.0 (8.5 - 11.5)	1.0 (0.0 - 1.0)	10.0 (9.0 - 11.0)	0.0 (0.0 - 1.0)	0.01
PW (mm)	10.0 (9.5 - 11.0)	1.0 (0.0 - 1.0)	10.0 (9.0 - 11.0)	0.0 (-1.0 - 1.0)	0.50
RV functions					
Tapse (mm)	23.5 (21.0 - 25.0)	-1.0 (-3.5 - 0.5)	24.5 (21.0 - 28.0)	-2.0 (-4.0 - 0.0)	0.63
RV s’ (cm/s)	11.3 (9.5 - 14.1)	-0.2 (-0.6 - 1.5)	12.0 (10.3 - 13.2)	-0.5 (-1.4 - 0.7)	0.34
RV Ee’ ratio	4.2 (3.3 - 5.0)	0.7 (-1.6 - 2.5)	3.9 (3.3 - 4.9)	0.1 (-0.3 - 0.8)	0.88
LV functions					
EF (%)	62.5 (60.0 - 64.5)	1.5 (-4.5 - 3.5)	62.0 (58.0 - 65.0)	0.0 (-4.0 - 4.0)	0.87
Mitral E (cm/s)	68.8 (58.5 - 79.0)	-4.3 (-6.0 - 0.3)	74.0 (64.7 - 84.8)	-3.8 (-10.8 - 3.9)	0.94
Dt (ms)	204 (192 - 236)	32 (9–64)	230 (208 - 261)	0 (-29 - 21)	0.008
EA ratio	0.8 (0.7 - 0.9)	0.0 (-0.1 - 0.1)	1.0 (0.8 - 1.1)	0.0 (-0.1 - 0.1)	0.99
IVRT (ms)	113 (89 - 122)	3 (-6 - 21)	103 (90 - 123)	4 (-10 - 21)	0.92
Ee’ ratio	8.9 (6.9 - 10.9)	0.6 (-2.0 - 1.5)	9.3 (7.4 - 10.6)	-0.3 (-1.4 - 0.6)	0.30

*LV* left ventricle, *LVEDD* left ventricular end diastolic diameter, *LVESD* left ventricular end systolic diameter, *IVS* interventricular septum thickness, *PW* thickness of left ventricle’s posterior wall, *RV* right ventricle, *tapse* tricuspid annular plane systolic excursion, *RV’s* systolic tissue doppler measurement of right ventricle’s free wall, *RV Ee’ratio* the ratio of early tricuspid inflow to annular diastolic velocity, *EF* ejection fraction, *Mitral E* first peak of diastole, active filling, *Dt* deceleration time during diastole, *EA ratio* ratio of diastolic peaks E and A, *IVRT* isovolumic relaxation time, *Ee’ratio* ratio of early transmitral flow velocity (E) to early diastolic velocity of the mitral valve annulus (e)

*p value between groups

### ECG

Normal sinus rhythms were present in all ECG recordings with no signs of atrial or ventricular abnormalities. RT caused moderate T-wave alterations in 3/12 (25 %) recordings among group A and 13/46 (28 %) among group B (*p* = 0.82).

## Discussion

We demonstrated that high sensitivity cardiac troponin T levels increased during the whole breast adjuvant RT in every fifth patient. In addition, the increase in the hscTnT release associated positively with cardiac radiation doses and with minor changes in LV’s measurements suggesting that RT caused subclinical myocardial damage.

### High sensitivity cardiac TnT release

Cardiac troponin T is a well-established biomarker of cardiac damage in myocardial infarction due to ischemic heart disease. Moreover, it can detect cardiac damage in other clinical conditions such as heart failure [16, 17] and LV hypertrophy [18, 19]. The hscTnT values in healthy individuals have varied in different studies. The 99th percentile differed from 12 ng/l [20] to 20 ng/l [21] and is largely shown to be influenced by patient’s age, gender and comorbidities [22]. By excluding patients with underlying cardiac diseases, it is estimated that the median values (IQR) of hscTnT for women <55 years as well as <75 years are <3 (<3 - < 3) ng/l and the upper normal 99th percentile limit is 3.4 ng/l for women <55 years and 11.4 ng/l for women <75 years [22]. The weekly within person variation of hscTnT in healthy individuals is estimated to be 8 % [23] -32 % [24]. Diurnal changes in hscTnT are also observed with a decline in values by 0.8 % per hour from 8:30 to 14:30 [23]. Based on these results, a relevant increase in hscTnT levels during RT was predefined to exceed 30 % from individual baseline in this study. In our patients, the serum samples at the baseline and at the end of the RT were taken in the morning within a 2 h maximum time difference in 55 patients. In the remaining three patients (all in the group B), the time difference was 2.1, 4 and 5 h. The hscTnT levels in the group A increased 40 %–325 % from baseline and, therefore, the change cannot be explained by normal weekly or diurnal variation.

In cancer patients, elevated troponin T and I levels have been reported after various chemotherapy regimens [25–27]. In addition, the elevation of TnT during or shortly after chemotherapy correlated with long term negative changes in echocardiography [25, 27]. The effects of adjuvant breast cancer RT on TnT levels were not detected in earlier studies with less sensitive troponin methods [28, 29]. In a study by Nellessen et al. [11], thoracic RT for breast or lung cancer in 23 patients increased troponin levels measured during and at the end of RT. It is, however, noteworthy that the majority (16/23) of these patients had received prior chemotherapy.

Our patients were, as described earlier, previously untreated by chemotherapy. Therefore, these observed changes represent the radiation induced damage to myocardium. The RT-induced harmful process in the heart is thought to evolve from the clotting of myocardial microvessels and the subsequent hypoxia leading to myocyte damage [12]. The absolute levels of hscTnT at the end of RT in our patients were below the clinically used threshold for myocardial infarction (50 ng/l). Nevertheless, measurable (>4 ng/l) troponin levels indicate worse cardiac long term prognosis in other clinical conditions [17, 18].

### Cardiac radiation doses

In the previous studies, the mean cardiac radiation dose has varied from 4.9 Gy (retrospective analysis) [4] to 9 Gy (prospective analysis) [5]. In our study, the mean heart dose in the group A was significantly higher (4.0 ± 1.8 Gy) than in the group B (2.8 ± 1.4 Gy). As presented in Table 2 and Fig. 2, also the measured dose-volume points for the whole heart were significantly different between the groups. In group A, larger volumes of the cardiac structures received more radiation. These findings show, that increasing cardiac doses associate with myocyte damage measured by hscTnT release. In animal models, even doses as low as 2 Gy for the whole heart induced cardiac remodelling and fibrinogenesis [30]. In a retrospective study on breast cancer patients, the risk of major cardiac event increased linearly with every increasing Gy for the heart [4]. Absolutely safe cardiac doses cannot be extrapolated either from our results or from previous data. Furthermore, it is still unclear, whether a low dose for a larger cardiac volume is more detrimental than a higher dose for a smaller volume. However, our DVH data suggest that if the mean dose for the whole heart is under 2 Gy at least the risk of acute myocyte damage is low. This can easily be reached in majority of node-negative patients, if vDIBH (own institutional experience) or Active Breathing Control (ABC) [31] is utilized.

Increasing doses to LV correlate with myocardial perfusion defects [6, 7, 32]. In our patients, the mean dose to LV was higher in the group A and the difference in V5 Gy, V10 Gy and V20 Gy was also significant.

The LAD runs in the sulcus between the left and right ventricle. Its anatomical location in relation to chest wall varies from one patient to another. The LAD is, however, generally at the most anterior part of the LV and therefore, the radiation doses to LAD in left sided breast cancer RT can be substantial. In coronary vessels, the acute RT induced damage may later manifest as thickening and fibrosis of the vessel walls and increased local atherosclerosis. The coronary arteries should be considered as a serial-type organ, where the damage to one small part affects the function of whole the unit. In our patients, the DVHs of the LAD differ clearly after 5 Gy and converge only at high doses. The AUC analysis of the LAD did not reach statistical significance due to variation of doses and the limited number of patients.

### Echocardiographic changes

LV’s ejection fraction (LVEF), a widely used marker of the LV’s systolic function in oncology, did not decrease in our patients after RT. This finding is similar than with Lo Q et al. [33], who detected subclinical cardiac dysfunction by 2-D strain imaging in LV after left-sided breast cancer RT, but LVEF remained unchanged. In addition, LVEF can remain within normal limits even in a presence of significant cardiac diastolic dysfunction. This entity of heart failure with preserved ejection fraction (HFpEF) is more common in elderly women and it is thought to have a multi-factorial etiology [34, 35]. It might share some pathophysiology with radiation induced heart disease such as increased fibrosis markers [36] and proinflammatory changes [37].

Diastolic changes in LV’s function were observed in both groups such as lower mitral E peak. As RT induces inflammation and later fibrinogenesis in tissues [12], it is logical that the early cardiac changes would be first present in the diastole, where the impaired relaxation and increased stiffness in ventricular walls cause impaired filling. The prolongation of diastolic deceleration time, a representative of increased ventricular wall stiffness, was apparent after RT in group A accompanied with increased septum thicknesses in contrast to group B. In addition, the LV diameters in both end systole and diastole decreased more in group A than group B- yet this failed to reach statistical significance due to small number of patients. Diastolic dysfunction can cause major cardiac morbidity such as dyspnea in mild exertion, fatigue and fluid retention [38]. These findings further support the value of troponin release as a simple marker of radiation-induced myocardial damage.

### Confounding factors and limitations

Hypertension is a known additive risk factor for RT induced cardiotoxicity [4]. In our patients, 50 % of the group A patients had hypertension compared with 30 % in the group B. However, this difference was not significant. It is of interest, that none of the patients using thyroid hormone supplementation had increases in hscTnT levels. There is experimental data on the benefits of thyroid hormone use after myocardial infarction (MI) in rodents [39, 40], and the beneficial effects of high T3 levels on cardiac functions after MI have been described also in humans [41]. This finding is intriguing and should be validated in a larger population as no reliable and clinically relevant subgroup analysis could be performed in our study due to the relatively small number of patients.

### Clinical implications

HscTnT is a sensitive marker of acute myocardial damage. In general, greater damage causes a greater troponin release and indicates worse prognosis. In this study, the increases in hscTnT values associated with increased cardiac radiation doses for the whole heart and LV. In daily RT treatment planning, at least the whole heart should be contoured as an organ at risk for left sided breast cancer RT in order to keep the cardiac dose as low as possible. Evolving radiation techniques will help to accomplish the cardiac dose sparing. Patient derived risk factors, such as hypertension, are still important and need special caution and guidance. Echocardiography can measure RT induced subclinical cardiac changes immediately after breast cancer RT and should be used in the later follow up of patients. Likewise, an expert consensus from 2013 recommends routine echocardiography after left sided breast cancer RT for all breast cancer survivors in 5–10 years to screen for radiation induced heart disease [42].

## Conclusions

The elevation on hscTnT levels suggests that adjuvant RT causes subclinical myocardial damage in patients with left-sided breast cancer. As the increase in hscTnT associates with increased cardiac radiation doses all efforts should be made to keep the radiation to the heart as low as possible. Whether these acute subclinical changes in echocardiography increase the risk of excessive long-term cardiovascular morbidity or mortality in our patients will be addressed in the later follow-up.

## References

[1] Darby S, McGale P, Correa C, Taylor C, Arriagada R, Clarke M, Cutter D, Davies C, Ewertz M, Godwin J, Gray R, Pierce L, Whelan T, Wang Y, Peto R, Early Breast Cancer Trialists’ Collaborative Group (EBCTCG) (2011). Effect of radiotherapy after breast-conserving surgery on 10-year recurrence and 15-year breast cancer death: meta-analysis of individual patient data for 10,801 women in 17 randomised trials. Lancet.

[2] Bardia A, Arieas ET, Zhang Z, Defilippis A, Tarpinian K, Jeter S, Nguyen A, Henry NL, Flockhart DA, Hayes DF, Hayden J, Storniolo AM, Armstrong DK, Davidson NE, Fetting J, Ouyang P, Wolff AC, Blumenthal RS, Ashen MD, Stearns V (2012). Comparison of breast cancer recurrence risk and cardiovascular disease incidence risk among postmenopausal women with breast cancer. Breast Cancer Res Treat.

[3] Bouillon K, Haddy N, Delaloge S, Garbay JR, Garsi JP, Brindel P, Mousannif A, Le MG, Labbe M, Arriagada R, Jougla E, Chavaudra J, Diallo I, Rubino C, de Vathaire F (2011). Long-term cardiovascular mortality after radiotherapy for breast cancer. J Am Coll Cardiol.

[4] Darby SC, Ewertz M, McGale P, Bennet AM, Blom-Goldman U, Bronnum D, Correa C, Cutter D, Gagliardi G, Gigante B, Jensen MB, Nisbet A, Peto R, Rahimi K, Taylor C, Hall P (2013). Risk of ischemic heart disease in women after radiotherapy for breast cancer. N Engl J Med.

[5] Erven K, Florian A, Slagmolen P, Sweldens C, Jurcut R, Wildiers H, Voigt JU, Weltens C (2013). Subclinical cardiotoxicity detected by strain rate imaging up to 14 months after breast radiation therapy. Int J Radiat Oncol Biol Phys.

[6] Gyenes G, Fornander T, Carlens P, Glas U, Rutqvist LE (1996). Myocardial damage in breast cancer patients treated with adjuvant radiotherapy: a prospective study. Int J Radiat Oncol Biol Phys.

[7] Marks LB, Yu X, Prosnitz RG, Zhou SM, Hardenbergh PH, Blazing M, Hollis D, Lind P, Tisch A, Wong TZ, Borges-Neto S (2005). The incidence and functional consequences of RT-associated cardiac perfusion defects. Int J Radiat Oncol Biol Phys.

[8] Prosnitz RG, Hubbs JL, Evans ES, Zhou SM, Yu X, Blazing MA, Hollis DR, Tisch A, Wong TZ, Borges-Neto S, Hardenbergh PH, Marks LB (2007). Prospective assessment of radiotherapy-associated cardiac toxicity in breast cancer patients: analysis of data 3 to 6 years after treatment. Cancer.

[9] Dogru A, Cabuk D, Sahin T, Dolasik I, Temiz S, Uygun K (2013). Evaluation of cardiotoxicity via speckle-tracking echocardiography in patients treated with anthracyclines. Onkologie.

[10] Florescu M, Magda LS, Enescu OA, Jinga D, Vinereanu D (2014). Early detection of epirubicin-induced cardiotoxicity in patients with breast cancer. J Am Soc Echocardiogr.

[11] Nellessen U, Zingel M, Hecker H, Bahnsen J, Borschke D (2010). Effects of radiation therapy on myocardial cell integrity and pump function: which role for cardiac biomarkers?. Chemotherapy.

[12] Stewart FA (2012). Mechanisms and dose–response relationships for radiation-induced cardiovascular disease. Ann ICRP.

[13] Feng M, Moran JM, Koelling T, Chughtai A, Chan JL, Freedman L, Hayman JA, Jagsi R, Jolly S, Larouere J, Soriano J, Marsh R, Pierce LJ (2011). Development and validation of a heart atlas to study cardiac exposure to radiation following treatment for breast cancer. Int J Radiat Oncol Biol Phys.

[14] Croghan CW, Egeghy EP: Methods of Dealing with Values Below the Limit of Detection using SAS. 2003 http://analytics.ncsu.edu/sesug/2003/SD08-Croghan.pdf

[15] Evangelista A, Flachskampf F, Lancellotti P, Badano L, Aguilar R, Monaghan M, Zamorano J, Nihoyannopoulos P, European Association of Echocardiography (2008). European Association of Echocardiography recommendations for standardization of performance, digital storage and reporting of echocardiographic studies. Eur J Echocardiogr.

[16] Takashio S, Yamamuro M, Uemura T, Utsunomiya D, Morita K, Izumiya Y, Sugiyama S, Kojima S, Yamamoto E, Tsujita K, Tanaka T, Tayama S, Kaikita K, Hokimoto S, Yasuda O, Yamashita Y, Ogawa H (2014). Correlation between extent of myocardial fibrosis assessed by cardiac magnetic resonance and cardiac troponin T release in patients with nonischemic heart failure. Am J Cardiol.

[17] Jungbauer CG, Riedlinger J, Buchner S, Birner C, Resch M, Lubnow M, Debl K, Buesing M, Huedig H, Riegger G, Luchner A (2011). High-sensitive troponin T in chronic heart failure correlates with severity of symptoms, left ventricular dysfunction and prognosis independently from N-terminal pro-b-type natriuretic peptide. Clin Chem Lab Med.

[18] Cramer G, Bakker J, Gommans F, Brouwer M, Kurvers M, Fouraux M, Verheugt F, Kofflard M (2014). Relation of highly sensitive cardiac troponin T in hypertrophic cardiomyopathy to left ventricular mass and cardiovascular risk. Am J Cardiol.

[19] Miao DM, Zhang LP, Yu HP, Zhang JY, Xiao WK, Ye P (2014). Serum levels of high-sensitivity troponin T: a novel marker for left ventricular remodeling and performance in hypertensive subjects. Genet Mol Res.

[20] Latini R, Masson S, Anand IS, Missov E, Carlson M, Vago T, Angelici L, Barlera S, Parrinello G, Maggioni AP, Tognoni G, Cohn JN, Val-HeFT Investigators (2007). Prognostic value of very low plasma concentrations of troponin T in patients with stable chronic heart failure. Circulation.

[21] Apple FS, Ler R, Murakami MM (2012). Determination of 19 cardiac troponin I and T assay 99th percentile values from a common presumably healthy population. Clin Chem.

[22] Koerbin G, Abhayaratna WP, Potter JM, Apple FS, Jaffe AS, Ravalico TH, Hickman PE (2013). Effect of population selection on 99th percentile values for a high sensitivity cardiac troponin I and T assays. Clin Biochem.

[23] Aakre KM, Roraas T, Petersen PH, Svarstad E, Sellevoll H, Skadberg O, Saele K, Sandberg S (2014). Weekly and 90-minute biological variations in cardiac troponin T and cardiac troponin I in hemodialysis patients and healthy controls. Clin Chem.

[24] Frankenstein L, Wu AH, Hallermayer K, Wians FH, Giannitsis E, Katus HA (2011). Biological variation and reference change value of high-sensitivity troponin T in healthy individuals during short and intermediate follow-up periods. Clin Chem.

[25] Lipshultz SE, Miller TL, Scully RE, Lipsitz SR, Rifai N, Silverman LB, Colan SD, Neuberg DS, Dahlberg SE, Henkel JM, Asselin BL, Athale UH, Clavell LA, Laverdiere C, Michon B, Schorin MA, Sallan SE (2012). Changes in cardiac biomarkers during doxorubicin treatment of pediatric patients with high-risk acute lymphoblastic leukemia: associations with long-term echocardiographic outcomes. J Clin Oncol.

[26] Sawaya H, Sebag IA, Plana JC, Januzzi JL, Ky B, Cohen V, Gosavi S, Carver JR, Wiegers SE, Martin RP, Picard MH, Gerszten RE, Halpern EF, Passeri J, Kuter I, Scherrer-Crosbie M (2011). Early detection and prediction of cardiotoxicity in chemotherapy-treated patients. Am J Cardiol.

[27] Cardinale D, Sandri MT, Martinoni A, Borghini E, Civelli M, Lamantia G, Cinieri S, Martinelli G, Fiorentini C, Cipolla CM (2002). Myocardial injury revealed by plasma troponin I in breast cancer treated with high-dose chemotherapy. Ann Oncol.

[28] D’Errico MP, Grimaldi L, Petruzzelli MF, Gianicolo EA, Tramacere F, Monetti A, Placella R, Pili G, Andreassi MG, Sicari R, Picano E, Portaluri M (2012). N-terminal pro-B-type natriuretic peptide plasma levels as a potential biomarker for cardiac damage after radiotherapy in patients with left-sided breast cancer. Int J Radiat Oncol Biol Phys.

[29] Hughes-Davies L, Sacks D, Rescigno J, Howard S, Harris J (1995). Serum cardiac troponin T levels during treatment of early-stage breast cancer. J Clin Oncol.

[30] Monceau V, Meziani L, Strup-Perrot C, Morel E, Schmidt M, Haagen J, Escoubet B, Dorr W, Vozenin MC (2013). Enhanced sensitivity to low dose irradiation of ApoE−/− mice mediated by early pro-inflammatory profile and delayed activation of the TGFbeta1 cascade involved in fibrogenesis. PLoS One.

[31] Swanson T, Grills IS, Ye H, Entwistle A, Teahan M, Letts N, Yan D, Duquette J, Vicini FA (2013). Six-year experience routinely using moderate deep inspiration breath-hold for the reduction of cardiac dose in left-sided breast irradiation for patients with early-stage or locally advanced breast cancer. Am J Clin Oncol.

[32] Lind PA, Pagnanelli R, Marks LB, Borges-Neto S, Hu C, Zhou SM, Light K, Hardenbergh PH (2003). Myocardial perfusion changes in patients irradiated for left-sided breast cancer and correlation with coronary artery distribution. Int J Radiat Oncol Biol Phys.

[33] Lo Q, Hee L, Batumalai V, Allman C, MacDonald P, Delaney GP, Lonergan D, Thomas L (2015). Subclinical cardiac dysfunction detected by strain imaging during breast irradiation with persistent changes 6 weeks after treatment. Int J Radiat Oncol Biol Phys.

[34] Poppe KK, Doughty RN (2014). Outcomes in patients with heart failure with preserved ejection fraction. Heart Fail Clin.

[35] Upadhya, B, Taffet, GE, Cheng, CP, Kitzman, DW. Heart failure with preserved ejection fraction in the elderly: scope of the problem. J Mol Cell Cardiol 2015;83:73–87. 10.1016/j.yjmcc.2015.02.025 PMC5300019 25754674

[36] Tromp J, van der Pol A, Klip IT, de Boer RA, Jaarsma T, van Gilst WH, Voors AA, van Veldhuisen DJ, van der Meer P (2014). Fibrosis marker syndecan-1 and outcome in patients with heart failure with reduced and preserved ejection fraction. Circ Heart Fail.

[37] Zuo, L, Chuang, CC, Hemmelgarn, BT, Best, TM : Heart failure with preserved ejection fraction: defining the function of ROS and NO. J Appl Physiol (1985) 2015 epub, ahead of print: jap.01149.2014. 10.1152/japplphysiol.01149.2014 25977452

[38] Sharma K, Kass DA (2014). Heart failure with preserved ejection fraction: mechanisms, clinical features, and therapies. Circ Res.

[39] Pantos C, Mourouzis I, Markakis K, Tsagoulis N, Panagiotou M, Cokkinos DV (2008). Long-term thyroid hormone administration reshapes left ventricular chamber and improves cardiac function after myocardial infarction in rats. Basic Res Cardiol.

[40] Pantos C, Mourouzis I, Markakis K, Dimopoulos A, Xinaris C, Kokkinos AD, Panagiotou M, Cokkinos DV (2007). Thyroid hormone attenuates cardiac remodeling and improves hemodynamics early after acute myocardial infarction in rats. Eur J Cardiothorac Surg.

[41] Lymvaios I, Mourouzis I, Cokkinos DV, Dimopoulos MA, Toumanidis ST, Pantos C (2011). Thyroid hormone and recovery of cardiac function in patients with acute myocardial infarction: a strong association?. Eur J Endocrinol.

[42] Lancellotti P, Nkomo VT, Badano LP, Bergler-Klein J, Bogaert J, Davin L, Cosyns B, Coucke P, Dulgheru R, Edvardsen T, Gaemperli O, Galderisi M, Griffin B, Heidenreich PA, Nieman K, Plana JC, Port SC, Scherrer-Crosbie M, Schwartz RG, Sebag IA, Voigt JU, Wann S, Yang PC, European Society of Cardiology Working Groups on Nuclear Cardiology and Cardiac Computed Tomography and Cardiovascular Magnetic Resonance, American Society of Nuclear Cardiology, Society for Cardiovascular Magnetic Resonance, and Society of Cardiovascular Computed Tomography (2013). Expert consensus for multi-modality imaging evaluation of cardiovascular complications of radiotherapy in adults: a report from the European Association of Cardiovascular Imaging and the American Society of Echocardiography. J Am Soc Echocardiogr.

